# Maximising Cancer Prevention and Early Detection in the Australian Cancer Plan: Where Are We Now and What Should Come Next?

**DOI:** 10.5694/mja2.70323

**Published:** 2026-10-31

**Authors:** Erin L. Symonds, Billie Bonevski, Richard Edwards, Nathan J. Harrison, Sana Ishaque, Nicole M. Rankin, Sibel Saya, Renee J. Smith, Carolyn Ee

**Affiliations:** ^1^ Flinders Health and Medical Research Institute, College of Medicine and Public Health Flinders University Adelaide South Australia Australia; ^2^ Gastroenterology Department, Flinders Medical Centre Southern Adelaide Local Health Network Adelaide South Australia Australia; ^3^ National Centre for Education and Training on Addiction, Flinders Health and Medical Research Institute, College of Medicine and Public Health Flinders University Adelaide South Australia Australia; ^4^ Melbourne School of Population and Global Health University of Melbourne Melbourne Victoria Australia; ^5^ Department of General Practice and Primary Care and Collaborative Centre for Cancer Genomic Medicine University of Melbourne Melbourne Victoria Australia; ^6^ Flinders LiveWell Cancer Research Centre, Caring Futures Institute, College of Health and Enablement Flinders University Adelaide South Australia Australia

**Keywords:** early detection of cancer, health services, health services research, population health

## Abstract

Cancer accounts for three in 10 deaths in Australia, yet many cases are preventable or can be detected at an early stage. Maximising cancer prevention and early detection is one of the six strategic objectives of the 10‐year Australian Cancer Plan. This perspective article reviews the current progress and challenges of reducing the cancer burden, along with future opportunities. It explores how to address modifiable risk factors, particularly tobacco smoking, alcohol use and sun exposure and the advances in screening programs for bowel, breast, cervical and lung cancers. It highlights the potential for risk‐stratified and genomics‐informed screening, emphasising the need to support participation through equitable and culturally safe approaches.

## 1 Introduction

In 2024, three in 10 deaths in Australia were attributable to cancer [1]. Almost half of the cancer burden in Australia is linked to modifiable risk factors, with global studies showing that 42% of cancer deaths and 38% of cancer cases are preventable [2, 3]. Within Australia, almost 230,000 cancer‐related deaths have been averted since the 1980s due to evidence‐based primary (e.g., tobacco control) and secondary (e.g., cancer screening) prevention measures [4].

This perspective article provides an overview of current progress and reviews evidence, challenges and opportunities for its strategic objective of maximising cancer prevention and early detection in Australia.

## 2 Reducing Modifiable Cancer Risk Factors

Key modifiable risk factors for cancer include tobacco smoking, alcohol use, sun exposure, overweight and obesity, diet, physical inactivity, occupational exposures and infections (e.g., human papillomavirus [HPV]), in addition to metabolic conditions (e.g., Type 2 diabetes). Tobacco use is the largest contributor (15.4% of total cancer burden in 2024 [5]), being associated with ≥ 10 cancers [6], and is the main aetiological factor for lung cancer, the commonest cause of cancer death in Australia [1]. Alcohol (3.6% of total cancer burden [5]) is a Group 1 carcinogen linked with ≥ 7 cancers [7], causing an estimated 5800 Australian cancer cases in 2020 [8]. Exposure to ultraviolet (UV) radiation from the sun (3.2% of total cancer burden [5]) is the main risk factor for melanoma and non‐melanoma skin cancers. Risk of some cancers also increases through interactive effects (e.g., between alcohol and smoking) [9]. We highlight these three risk factors because of their well‐established causal role in cancer and because they represent the most mature examples of effective population‐based prevention interventions. However, additional prevention efforts would make a substantial contribution to cancer prevention in Australia, given the proportion of total cancer burden attributable to other risk factors, particularly reducing overweight and obesity (6.0% of burden), improving diet (3.3% of burden) and increasing physical activity (1.2% of burden) [10].

### 2.1 Progress and Challenges

A lens for assessing progress, challenges and opportunities for maximising cancer prevention in Australia is the degree to which the principles and approaches of the Ottawa Charter for health promotion [11] have been implemented [12]. Adopted in 1986, the Charter includes five key actions: building healthy public policy, creating supportive environments, strengthening community actions, developing personal skills and reorienting health services.

The National Preventive Health Strategy targets reducing smoking prevalence and alcohol consumption [13] and outlines strategies for control. Sixty‐nine per cent of Australians have consumed alcohol in the previous 12 months, 32% in excess of national guidelines [14]. Yet, there is limited public understanding of alcohol's carcinogenicity [15]. A recent review of the National Alcohol Strategy concluded there has been minimal change in alcohol use and harms since 2019 [16]. Implementation of public policies for alcohol control is limited and measures such as health warning labels have attracted significant industry opposition [17]. Price‐based policies in the Northern Territory were repealed despite favourable evidence for reducing alcohol consumption and harms [18].

In contrast, Australia has been a leader in tobacco control through introducing policies such as advertising bans, substantial excise increases and plain packaging of cigarettes [19]. Daily tobacco smoking prevalence more than halved between 2001 and 2022–2023 (from 19.4% to 8.3%); however, modelling suggests that the goal of overall national prevalence of 5% by 2030 will not be achieved [20]. Recently mandated health‐promoting pack inserts, world‐leading warning labels on cigarette sticks and removal of additives such as menthol have been implemented [19]. However, more robust endgame interventions, such as a generational ban on tobacco sales, restricting tobacco retailer supply and reducing nicotine content, have not progressed despite a commitment to explore these options in the National Tobacco Strategy [21]. There is also growing concern about the extent of the illicit tobacco and e‐cigarette market [22], leading to some experts calling for tobacco taxes to be reduced or further increases paused. Similar calls have also been made by the tobacco industry, highlighting the ongoing challenge of industry interference in tobacco control policy [23]. This challenge is also relevant to other risk factors influenced by commercial determinants of health (e.g., alcohol and ultra‐processed food industries).

Australia is an international leader in implementing policies to reduce UV exposure by banning commercial solaria in 2015, introducing sales tax exemptions for sunscreen products and creating supportive environments by requiring sun protection procedures and provision of shade in education and care provider settings [24, 25]. There is a long history of mass media campaigns and supportive community action to increase knowledge of the link between harmful sun exposure and skin cancer and promote uptake of sun protection behaviours, including the ‘Slip, Slop, Slap!’ campaign (later ‘Slip, Slop, Slap, Seek, Slide’) [26]. However, the incidence of skin cancer, including melanoma, remains unacceptably high and costs the Australian health economy $1.7 billion a year [27]. An opportunity for strengthening policy and practice is prioritising skin cancer in the National Preventive Health Strategy [13] and considering a dedicated prevention strategy. This builds on sustained government investment, including initiatives through Cancer Council Australia such as the SunSmart program and the ‘Save Your Skin’ campaign. Evidence demonstrates cost‐effectiveness of key prevention measures with a two‐ to fourfold return on investment [27]. Recent reports that several sunscreens on the Australian market did not demonstrate their advertised level of sun protection may have impacted public trust in sunscreen effectiveness, creating an opportunity to reinforce the importance of sunscreen application and other risk‐reducing behaviours.

### 2.2 Opportunities and Priorities for Action

Using the Ottawa Charter framework priorities to address modifiable risk factors will include further strengthening health education and community action, implementing additional healthy public policies and continuing to create supportive environments. The intervention priorities described below are consistent with those specified in evidence‐based frameworks to reduce harms, including from the World Health Organization: the INTERSUN programme for UV [28], the SAFER initiative for alcohol [29] and the forward‐looking tobacco control measures [30].

For UV reduction, these should include increased investment in mass media campaigns, implementing mandatory sun protection policies in secondary schools and greater provision of shade in public places. Consistent with the SAFER initiative, reducing alcohol promotion and availability and increasing the price of alcohol (e.g., via volumetric taxation) [31] would support reducing alcohol‐related cancers and other harms. For tobacco smoking, the priority should be bold forward‐looking measures [30], such as the endgame interventions described in the previous section to ensure that the national goal of 5% prevalence by 2030 is achieved rapidly and equitably across all population groups. These should be accompanied by robust measures to tackle illicit trade such as adoption of a track and trace system and greatly enhanced enforcement [32].

Infection control is another example of reducing cancer risk. Persistent infection with HPV is linked to at least six cancer types. With a strategy of 90% HPV vaccination coverage, Australia has aligned with the World Health Organization's goals of eliminating cervical cancer, with these targets within reach by 2030 [33]. Further prevention of cancer could be achieved through healthy public policies and intensive health promotion and community‐based interventions to address other modifiable risk factors such as diet and physical activity.

## 3 Promoting and Expanding Screening and Early Detection Programs

For several cancers, screening not only allows for early detection but also prevention through the identification and removal of pre‐cancerous lesions. Australia has had population‐based national screening programs since 1991 when BreastScreen Australia and the National Cervical Screening Program were introduced. BreastScreen Australia involves biennial mammograms for women aged 50–74 years, with women aged 40–49 also eligible to attend. The National Cervical Screening Program began with 2‐yearly Papanicolaou smear tests and evolved to a 5‐yearly HPV test for people with a cervix aged 25–74 years in 2017, with self‐collection introduced in 2022 [10]. The National Bowel Cancer Screening Program commenced in 2006, with full implementation by 2019 of biennial immunochemical faecal occult blood tests (iFOBT; also known as faecal immunochemical tests [FIT]) for individuals aged 50–74 years [34]. Eligibility was expanded to include 45‐ to 49‐year‐olds in 2024 [35] in response to rising incidence of early‐onset colorectal cancer [36]. The National Lung Cancer Screening Program was introduced in 2025 as a risk‐based program using low‐dose computed tomography scans to screen for lung cancer in people aged 50–70 years who currently smoke or have quit smoking in the past 10 years, with a ≥ 30 pack year history of smoking [37].

### 3.1 Progress and Challenges

The programs are supported by strong evidence of effectiveness; for example, a reduction of breast cancer mortality by 41% [38] and colorectal cancer mortality by 62% [39]. Lung cancer screening has been associated with a 24% reduction in cancer‐specific death [40], while HPV‐based screening provides up to 70% greater protection against cervical cancer compared with cytology [41]. Despite this, screening participation remains suboptimal in BreastScreen Australia (52%) [42] and in the National Bowel Cancer Screening Program (42%) [34], compared with cervical cancer screening (78%) [43]. A study found that, among screening non‐participants, 74% and 48% reported undergoing screening outside of BreastScreen Australia and the National Bowel Cancer Screening Program, respectively [44], indicating that true participation rates are likely under‐estimated. This highlights limitations in the current data collection, as BreastScreen Australia is not fully integrated into the National Cancer Screening Register, and screening outside of programs is not consistently recorded. Strengthening data capture for *all* screening will improve the accuracy of participation estimates and support more accurate modelling and evaluation.

Screening program design and delivery can limit the impact. Studies supported that National Bowel Cancer Screening Program eligibility should start earlier to address the growing incidence of early‐onset colorectal cancer and that the screening age should begin 5 years earlier for Aboriginal and Torres Strait Islander people to improve equity [45]. Yet eligibility for all individuals is currently restricted to people aged 45 years and above [35], with 45‐ to 49‐year‐olds having to actively opt into the program. Similarly, effectiveness of the National Lung Cancer Screening Program may be limited through the program design of targeting high‐risk populations.

There are calls to move towards more personalised or risk‐based screening. The Australian Cancer Plan (https://www.canceraustralia.gov.au/australian‐cancer‐plan) includes a 5‐year goal to ‘implement new, and improve existing, evidenced‐based, risk‐stratified cancer screening programs’. The 2026 BreastScreen Australia Strategic Framework [46] outlines a strategic goal of leading the way in high‐quality screening using technology and personalised risk‐stratified approaches. Moves towards personalised screening, however, are hampered by lack of evidence of effectiveness, which risk factors should inform risk‐stratified screening and how risk assessment can be delivered. Although there have been efforts to move towards more personalised approaches for breast cancer screening (e.g., reporting of breast density [47]), there is limited randomised controlled trial evidence on risk‐stratified screening [48]. The capacity of BreastScreen Australia centres to deliver new technologies such as digital breast tomosynthesis and magnetic resonance imaging scans presents another challenge. Health system fragmentation across jurisdictions limits the ability to examine national data to drive innovation and policy change [47, 49].

### 3.2 Opportunities and Priorities for Action

Opportunities for increasing early detection of cancers in Australia include reorienting health services to improve the delivery and uptake of current screening programs and developing new approaches such as the adoption of targeted or personalised screening strategies.

One priority is to further expand the National Cancer Screening Register to capture cancer screening undertaken outside of population‐based programs and improve its integration with primary care electronic medical records to better facilitate identification of patients eligible for screening [50]. In addition, aligning the National Cancer Screening Register with state‐based cancer registries would enhance data linkage across the cancer care continuum, supporting more comprehensive monitoring of outcomes, including stage‐specific reporting to assess early detection and inform service planning. This should be accompanied by the systematic implementation of evidence‐based approaches to equitably enhance screening uptake.

Population‐based screening programs should be regularly monitored and reviewed, particularly as population demographics change and new evidence emerges regarding population‐specific disease risks. Recent evidence suggests that prostate‐specific antigen screening can reduce prostate cancer deaths [51], while updated prostate cancer guidelines identify high‐risk groups, including Black males of sub‐Saharan ancestry aged over 45 years [52]. These developments highlight the importance of continually evaluating emerging evidence to determine whether changes to existing screening programs or the introduction of new national screening programs are warranted. Similarly, new technologies (e.g., multicancer early detection tests [53]) should be monitored to determine their potential role in population‐based screening programs. The benefits from screening must outweigh the potential harms from false‐positive results and over‐diagnosis.

Risk‐based screening approaches could address this and should continue to be reviewed including the availability of validated risk models and optimal risk stratification approaches. Risk models may incorporate socio‐economic determinants, behavioural and genomic risk factors, comorbidities and information from previous screening rounds. Tailored programs may consider risk factors unique to different priority populations, differences in biological predisposition to cancer or results from previous screening tests, such as FIT haemoglobin concentrations in colorectal cancer screening [54]. Emerging models are being assessed, including a large trial within BreastScreen Victoria assessing the safety and efficacy of artificial intelligence cancer detection using mammography images [55]. These have the potential to inform the development of personalised rescreening intervals and enable redistribution of resources towards under‐screened or priority populations.

Current Australian efforts to generate new evidence for risk‐based screening include the ‘Roadmap for a National Targeted Skin Cancer Screening Program’ [56], which is focused on melanoma and will identify how risk‐based screening should be delivered (e.g., selecting appropriate tools, defining the high‐risk population, testing interventions) to optimise the balance between benefits and harms. A ‘Roadmap to Liver Cancer Control in Australia’ similarly outlines key priorities for prevention and early detection of hepatocellular carcinoma, including implementing national hepatitis B and C strategies and establishing a national hepatocellular carcinoma surveillance system [57]. Local efforts can draw on international examples that seek to optimise the use of limited resources, such as in China, where a risk‐adjusted approach to lung, colorectal, liver, breast and nasopharyngeal cancer screening has been adopted, identifying high‐risk individuals by questionnaires, with or without pre‐screening rapid tests such as FIT or Epstein–Barr virus serology [58].

Incorporation of genomic factors, such as polygenic risk scores, are growing in their ability to stratify the population for tailored screening for common cancers such as breast [59], colorectal [60] and prostate cancer [61]. Internationally, significant efforts are underway to develop polygenic risk scores that encompass the diversity across ancestries [62] and there are initiatives to validate these scores in the Australian population [63]. However, before implementation, a careful review of the feasibility and evidence of potential risks and benefits is required [64].

## 4 Achieving Equity

The Australian Cancer Plan prioritises achieving equity in cancer control, in recognition of the significant disparities in exposure to risk factors for cancer and participation and outcomes of early detection programs. Many of these can be attributed to unequal distribution of social, cultural, commercial and environmental determinants of health [65], and how they overlap. Large inequities in smoking prevalence persist, with prevalence of 20.1% and 13.4% in Aboriginal and Torres Strait Islander and people living with the greatest socio‐economic disadvantage, respectively, in 2022–2023 and high prevalence also in the lesbian, gay, bisexual, transgender, queer, intersex and asexual community [10]. There are significant disparities in cancer screening participation across priority groups including in people living with mental illness [66], culturally and linguistically diverse populations [10], socio‐economically disadvantaged communities, rural/remote Australians, Aboriginal and Torres Strait Islanders and those living with disability [67]. Barriers to participation include concerns about downstream costs, lack of culturally tailored information, stigma, mistrust and lack of culturally safe healthcare services [68], whereas people living with mental illness report that cancer screening is rarely asked about by care providers [66].

Strategies to overcome participation barriers include personalised invitations and information materials, translated and culturally adapted for culturally and linguistically diverse communities and in Easy Read and video formats for people living with disability [68]. Health service and system improvements including education for healthcare providers and improving access via mobile screening vans are also effective [69].

Strengthening the policy and regulatory environment in cancer prevention and early detection must go beyond changing individual risk exposures or screening participation [70] and must also address the deeper structural social, commercial, political and digital determinants of health. It must also be recognised that screening is just the first step in the cancer care pathway, with inequities in access to timely diagnostic assessment, treatment and follow‐up care remaining a significant barrier to achieving equitable health outcomes [71]. These efforts should leverage community strengths; for example, patient navigators have emerged as an effective and cost‐effective way to improve prevention and early detection of cancer [72], particularly among priority populations.

Solutions that are developed with and led by community are a high priority. Policy should be informed by suitable approaches, such as community outreach [73, 74] and co‐design [75]. Exemplars include co‐design of the National Lung Cancer Screening Program with Aboriginal and Torres Strait Islander communities and informational resources tailored to several priority groups [76]. Portable, same‐day cervical screening and follow‐up care outreach programs in remote Western Australia were found to be feasible, with high levels of participant satisfaction [76].

Ensuring equity of access to and uptake of personalised screening is a key challenge, particularly given the limited availability of data needed for accurate risk stratification, such as genomic data for many ethnic groups.

## 5 Conclusion

Much has been achieved in cancer prevention over recent decades in Australia. However, there remains a significant burden from preventable cancer together with unacceptable health disparities in risk factor exposure, screening participation and early detection. We suggest investment and research priorities for action in Tables 1 and 2. Further efforts to provide evidence‐based prevention for all Australians include intensifying tobacco control efforts to achieve the 2030 goal of minimal smoking prevalence for all Australians and greatly strengthening preventive measures for other key cancer risk factors. Culturally safe, equitable and person‐centred access to cancer screening must be prioritised, including, where appropriate, targeted and risk‐based strategies for priority populations. Cancer screening programs provide an important opportunity to integrate primary prevention strategies [77]. Incorporating lifestyle risk assessment, education and support for behaviour change alongside screening could extend the benefits of screening beyond early detection. Approaches should be supported by appropriate investment in infrastructure, health system and data integration and workforce capability.

**TABLE 1:** Proposed investment priorities for cancer prevention and early detection.

Context and rationale	Investment priority	Perspectives for key groups and end users	Forward looking statement
Gaps in population‐based policy measures to address modifiable risk factors for cancer	Development and implementation of key policies including restricting alcohol supply and marketing, greater provision of shade in public places and secondary schools and bold endgame tobacco control measures ACP actions: 1.2.2; 1.5.1	Addressing key risk factors will improve population health, particularly for disadvantaged communities most impacted by cancer	Policy implementation will make Australia a world leader in reducing exposure to modifiable risk factors for cancer
Gaps in awareness of modifiable risk factors for cancer	Increase awareness of cancer risks and importance of reducing exposure (e.g., tobacco smoking, alcohol consumption, sun exposure) through mass media campaigns and community‐led programmes ACP actions: 1.2.1; 1.2.2; 1.5.1	Lowers cancer incidence and improves population health particularly for disadvantaged communities	Success will be assessed through public knowledge and reduced cancer incidence and deaths
Gaps in screening participation and outcome data	Expand the National Cancer Screening Register to capture all cancer screening participation and outcome data (including BreastScreen Australia and bowel cancer screening outside of the NBCSP) and pathology data, including cancer staging Identify and prioritise data gaps and strengthen data linkage to incorporate missing data into population‐based cancer registries ACP actions: 1.2.3; 1.2.4; 1.5.3	Accurate participation data will inform general practitioners on patient screening status Supports policymakers with evidence to guide screening programme design, resource allocation and eligibility criteria to maximise population health impact	Comprehensive information will enable more accurate programme monitoring and evaluation, cost‐effectiveness modelling and inform strategies to increase screening uptake
Inequitable participation in cancer screening, including for some high risk and disadvantaged populations	Leverage the impact of clinician recommendations on screening uptake by embedding screening strategies across all healthcare settings Co‐design and implement strategies to improve equity in screening Undertake feasibility assessments of risk‐stratified screening approaches, including evaluation of risks and benefits. ACP actions: 1.2.1; 1.2.4; 1.5.3; 1.5.4	Improves population health outcomes for currently under‐screened groups Supports guideline development by enabling implementation of high‐priority risk‐stratified screening approaches that incorporate evidence on risk factors, to ensure recommendations are appropriate for different priority populations	Equitable cancer detection at a more treatable stage will be enabled by enhanced data systems, workforce training, community engagement and consumer‐informed strategies Enhanced implementation of risk‐based screening and integration of genomics will lead to improved early detection and more efficient use of resources

*Note:* ACP Action 1.2.1: Cancer prevention and health promotion activities; ACP Action 1.2.2: Strengthen cancer prevention in broader health strategies; ACP Action 1.2.3: Translational research on determinants of health; ACP Action 1.2.4: Ongoing assessment of evidence for risk‐based screening; ACP Action 1.5.1: Strengthen policy and regulatory environment for modifiable risk factors and determinants of health; ACP Action 1.5.3: Implement new and improved evidenced‐based risk‐stratified screening programs; ACP Action 1.5.4: Genomics policy framework for cancer control.

Abbreviations: ACP, Australian Cancer Plan; NBCSP, National Bowel Cancer Screening Program.

**TABLE 2:** Proposed research priorities for cancer prevention and early detection.

Context and rationale	Research priority	Perspectives for key groups and end users	Forward looking statement
Significant inequities in cancer screening participation. Need for improved targeting of screening for priority populations, young adults and those with less common and rare cancers	Analysis of linked population screening, cancer registry and demographic data to identify gaps in early detection, complemented by co‐designed qualitative studies to understand barriers to access and participation Develop, pilot and implement targeted, risk‐informed screening initiatives ACP actions: 1.2.4; 1.5.3; 1.5.4	Will draw on end‐user experience and provide clinicians with tools to enhance participation, improve population health and decrease health costs to society	Will inform the evaluation of risk‐stratified and culturally appropriate screening, leading to increased participation, early detection and improved survival
Navigators and community outreach have emerged as effective approaches to promote equitable screening participation but have not been implemented at scale in Australia	Co‐designed strategies to improve participation in screening programmes for priority populations, including community‐based outreach models, lay/peer and professional navigator programmes and culturally adapted invitation and information systems Evaluation of digital screening delivery models for reaching under‐screened populations, including integration with primary care electronic medical records for opportunistic screening identification and scaling up of innovative models such as same‐day cervical screening and follow‐up care outreach programs ACP actions: 1.2.1; 1.2.3; 1.5.3	Supports health system, primary care services and community by generating evidence on effective, co‐designed implementation strategies to improve participation in screening for priority populations	Success measured by increased screening participation rates and reduction in late‐stage diagnosis rates across CALD, rural/remote and lower socio‐economic populations
To advance personalised screening approaches, risk‐based prediction tools, algorithms and polygenic risk scores need to be optimised	Determination of optimal risk prediction tools, algorithms and polygenic risk scores for the Australian population using linked data Implementation trials evaluating the safety, efficacy and cost‐effectiveness of risk‐stratified screening approaches, including in priority populations under‐represented in genomic data sets ACP actions: 1.2.4; 1.5.3; 1.5.4	Provides evidence to support shared decision‐making between clinicians and patients on personalised screening Informs health systems on cost‐effectiveness and equity impacts to inform decision as about new infrastructure for risk‐stratified screening	Success measured by adoption of risk prediction tools in primary care, alongside adoption of risk‐informed screening; equitable access to genomic risk assessment; reduction in interval cancers and over‐screening in low‐risk individuals More cancers are detected early, with health system cost savings and efficiencies
Major prevention interventions are often undertaken without robust evaluation	Systematic, cross‐disciplinary, rigorous research to evaluate the impact and effectiveness of prevention intervention, services and policies, including equity impacts ACP actions: 1.2.2; 1.2.3; 1.5.1	Research will inform priorities for health service and public health investment, and efficient use of resources	Will create a robust system for ongoing evidence generation to provide evidence for the implementation of effective, cost‐effective and pro‐equity preventive interventions in Australia and more widely

*Note:* ACP Action 1.2.1: Cancer prevention and health promotion activities; ACP Action 1.2.2: Strengthen cancer prevention in broader health strategies; ACP Action 1.2.3: Translational research on determinants of health; ACP Action 1.2.4: Ongoing assessment of evidence for risk‐based screening; ACP Action 1.5.1: Strengthen policy and regulatory environment for modifiable risk factors and determinants of health; ACP Action 1.5.3: Implement new and improved evidenced‐based risk‐stratified screening programs; ACP Action 1.5.4: Genomics policy framework for cancer control.

Abbreviations: ACP, Australian Cancer Plan; CALD, culturally and linguistically diverse.

## Author Contributions

**Erin L. Symonds:** conceptualisation, writing – original draft, writing – review and editing. **Billie Bonevski:** conceptualisation, writing – original draft, writing – review and editing. **Richard Edwards:** conceptualisation, writing – original draft, writing – review and editing. **Nathan J. Harrison:** conceptualisation, writing – original draft, writing – review and editing. **Sana Ishaque:** conceptualisation, writing – original draft, writing – review and editing. **Nicole M. Rankin:** conceptualisation, writing – original draft, writing – review and editing. **Sibel Saya:** conceptualisation, writing – original draft, writing – review and editing. **Renee J. Smith:** conceptualisation, writing – review and editing. **Carolyn Ee:** conceptualisation, writing – original draft, writing – review and editing.

## Funding

This article is part of a *Medical Journal of Australia* Collection on the Australian Cancer Plan and is funded by Flinders University. This work did not receive any other specific grant from funding agencies in the public, commercial or not‐for‐profit sectors.

## Disclosure

Not commissioned; externally peer reviewed.

## Conflicts of Interest

Carolyn Ee is Director of the Primary Care Collaborative Cancer Clinical Trials group, which is funded by a Cancer Australia Support for Cancer Clinical Trials program grant, and was an Expert Advisory Group member for the BreastScreen Australia National Policy and Funding Review (2024). Cancer Australia was given the opportunity to review this manuscript for factual accuracy. Cancer Australia had no role in the conception, design, analysis, interpretation or writing of the manuscript and had no influence over the content or conclusions. The authors have no other potential conflicts of interest to disclose.

## Data Availability

This article includes no original data.
